# Cosmetic emulsions containing innovative complex coacervates: A cross‐sectional study

**DOI:** 10.1111/ics.13035

**Published:** 2024-12-23

**Authors:** Delaporte Adeline, Grisel Michel, Gore Ecaterina

**Affiliations:** ^1^ Normandie Univ, URCOM UR 3221 Université Le Havre Normandi Le Havre France

**Keywords:** complex coacervation, emulsions, encapsulation, polymers, stability, α‐tocopherol

## Abstract

**Objective:**

Vitamin E, in the form of α‐tocopherol (TOCO), is an essential lipophilic antioxidant widely used in topical formulations. However, incorporating pure TOCO into skincare products poses significant challenges due to its limited solubility and high sensitivity to heat, light and oxidation. The present cross‐sectional study aimed to innovate by encapsulating TOCO using non‐animal sustainable biopolymers through complex coacervation and to investigate the interaction of these coacervates with cosmetic emulsions, focusing on their impact on the emulsions' physicochemical properties and stability.

**Methods:**

TOCO was encapsulated using the complex coacervation technique by combining two biopolymers: fungal chitosan and gum Arabic. The designed microcapsules were incorporated into oil‐in‐water emulsions containing natural ingredients, and the physicochemical properties as well as the stability of the formulations were evaluated and compared to those of non‐encapsulated TOCO emulsions.

**Results:**

Innovative coacervates of the non‐animal TOCO complex of 86.8 ± 3.5 μm were developed, achieving a high encapsulation efficiency and loading of 87.0% and 27.2%, respectively. The microcapsules exhibited thermal stability up to a temperature of 220°C and showed improved storage stability of the active ingredient when encapsulated. In particular, 63% of TOCO was retained over 2 months at a temperature of 40°C. Emulsions containing microcapsules showed increased particle size distribution, higher viscosity, and enhanced viscoelastic properties, in accordance with their textural properties. Both emulsions remained stable for a 1‐month storage period at a temperature of 40°C, and no noticeable effect of coacervates on the stability of TOCO in the emulsions was observed.

**Conclusion:**

This study emphasises the potential of fungal chitosan‐gum Arabic coacervates as a sustainable substitute for animal‐derived coacervates, demonstrating promising outcomes for the encapsulation of lipophilic actives. When incorporated into cosmetic emulsions, these coacervates enhanced the textural and rheological properties while preserving the TOCO stability over time. These findings suggest that the developed microcapsules offer considerable potential for the development of future skin‐care products with enhanced functional properties.

## Graphical abstract

This study demonstrates the successful encapsulation of α‐tocopherol using non‐animal biopolymers, improving its stability and functional properties in cosmetic emulsions. The developed microcapsules enhance product texture and rheological behaviour, offering promising potential for more stable and functional skincare formulations.

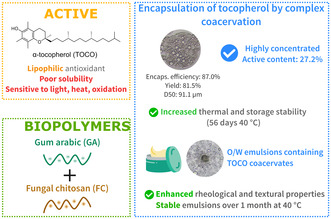

## Résumé

**Objectif:**

La vitamine E, sous forme de α‐tocophérol (TOCO), est un antioxydant lipophile essentiel largement utilisé dans les formulations topiques. Cependant, l’incorporation de TOCO pur dans les produits de soin de la peau pose des défis importants en raison de sa solubilité limitée et de sa forte sensibilité à la chaleur, à la lumière et à l’oxydation. Cette étude transversale visait à innover en encapsulant le TOCO à l’aide de biopolymères durables et non animaux via la coacervation complexe et à examiner l’interaction de ces coacervats avec des émulsions cosmétiques, en se concentrant sur leur impact sur les propriétés physico‐chimiques et la stabilité des émulsions.

**Méthodes:**

Le TOCO a été encapsulé par la technique de coacervation complexe, en combinant deux biopolymères : le chitosane d’origine fongique et la gomme arabique. Les microcapsules obtenues ont été incorporées dans des émulsions huile‐dans‐eau contenant des ingrédients naturels. Les propriétés physico‐chimiques et la stabilité des formulations ont été évaluées et comparées à celles d’émulsions contenant du TOCO non encapsulé.

**Résultats:**

Des coacervats innovants du complexe TOCO non animal, d’une taille moyenne de 86,8 ± 3,5 μm, ont été développés, avec une efficacité et une capacité d’encapsulation élevées de 87,0 % et 27,2 %, respectivement. Les microcapsules ont montré une stabilité thermique jusqu’à une température de 220 °C et une meilleure conservation de l’actif encapsulé, avec 63 % du TOCO retenu après 2 mois à 40 °C. Les émulsions contenant des microcapsules présentaient une augmentation de la distribution de taille des particules, une viscosité plus élevée et des propriétés viscoélastiques renforcées, en cohérence avec leurs propriétés texturales. Les deux types d’émulsions sont restées stables pendant 1 mois de stockage à 40 °C, et aucun effet notable des coacervats sur la stabilité du TOCO dans les émulsions n’a été observé.

**Conclusion:**

Cette étude met en avant le potentiel des coacervats chitosane fongique‐gomme arabique comme substitut durable aux coacervats d’origine animale. Les résultats montrent des perspectives prometteuses pour l’encapsulation d’actifs lipophiles. Lorsqu’ils sont intégrés dans des émulsions cosmétiques, ces coacervats améliorent les propriétés texturales et rhéologiques tout en préservant la stabilité du TOCO dans le temps. Ces microcapsules offrent un potentiel considérable pour le développement futur de produits de soin de la peau aux propriétés fonctionnelles renforcées.

## INTRODUCTION

α‐Tocopherol, the most important form of vitamin E, has been widely used for more than five decades in dermatological products due to its photoprotective properties [1]. This compound acts both as an antioxidant, due to its aromatic hydroxyl group, and as an anti‐inflammatory agent, reducing erythema and sunburn, while also acting as an anti‐wrinkle active [2, 3]. Topical application of α‐tocopherol enhances skin elasticity, structure, and overall appearance by firming and hydrating both the epidermis and dermis layers. However, its incorporation into skincare formulations is limited by its limited solubility and sensitivity to heat, light, and oxidation [4, 5]. To overcome these challenges, encapsulation techniques have been increasingly used to improve its stability and efficacy [5]. Microencapsulation involves the entrapment of a core (solid, liquid, or gas) within a polymeric matrix and may enhance its active properties, including its release, bioavailability, and stability. Several studies reported the application of microencapsulation for the protection of vitamin E, using techniques such as phase separation methods [6, 7], liposomes [8, 9], spray drying [10, 11], solid lipid nanoparticles (SLN) [12, 13] or spray cooling [14]. However, the potential future applications of these techniques in cosmetic formulations have not yet been investigated.

Complex coacervation is a highly effective microencapsulation method, involving the association of polyelectrolytes with opposite charges, resulting in phase separation and the formation of a coacervate phase surrounding the active compound [15]. The gelatin/gum Arabic system is one of the most studied coacervation systems described in the literature [16]. Nevertheless, since gelatin is produced from animal sources, there is a growing demand to explore non‐animal sustainable alternatives for developing stable and effective systems.

Gum Arabic, extracted from *Acacia Senegal*, is a negatively charged polysaccharide known for its high solubility and emulsifying properties [17, 18]. Chitosan, on the other hand, is a positively charged polysaccharide obtained by deacetylation of chitin from either fungus cell walls or crustacean shells. It has several advantageous characteristics, including biocompatibility, antioxidant and antimicrobial activity, and film formation properties [19, 20]. Fungal chitosan offers several benefits, such as being allergy‐friendly and vegan‐friendly with lower molecular weight, being free of heavy metals, and showing a higher degree of deacetylation. By reducing dependence on animal‐derived sources, it presents a more environmentally friendly alternative for the design of future skincare formulations [20]. However, the complex coacervation of fungal chitosan and gum Arabic is only poorly described [21, 22] and the impact on cosmetic systems remains uninvestigated. Furthermore, to our knowledge, no studies have investigated the combination of fungal‐chitosan and gum Arabic for the encapsulation of α‐tocopherol.

Although microencapsulation strategies of compounds are increasingly examined in the literature, only a few have discussed the incorporation of microcapsules in emulsions. Several studies incorporated microcapsules directly into commercially available cosmetic creams without specifying their complex composition. These studies investigated their stability mainly by assessing factors such as pH, organoleptic properties, and homogeneity [23, 24]. Khalid et al. [25] encapsulated fluconazole by complex coacervation with chitosan and chondroitin sulfate. Nanoparticles were introduced into water‐in‐oil (W/O) emulsions, and the permeation analysis demonstrated a greater retention of the enclosed active substance in comparison to a solution containing the active substance. Ang et al. [26] introduced curcuminoid coacervates in oil‐in‐water (O/W) emulsions, but the findings indicated that there was no notable improvement in the effectiveness of these emulsions. Böger et al. [27] incorporated roasted coffee oil coacervates into O/W emulsions without affecting the viscosity, consistency, and pH. They observed a reduction in the viscosity, spreadability, and adhesiveness of the product by instrumental measurements; however, these changes were not perceived during standardised sensory analysis methods. Moreover, the impact of the addition of microcapsules on the physicochemical properties of emulsions and their evolution over time during storage was not investigated.

This innovative cross‐sectional study investigates the development of a novel skincare formulation incorporating α‐tocopherol (TOCO), emphasising its encapsulation and subsequent integration into cosmetic matrices. Non‐animal bio‐based complex coacervates of TOCO were prepared by combining two biopolymers, namely fungal chitosan (FC) and gum Arabic (GA), through electrostatic interactions. TOCO was dissolved in diisopropyl adipate (DIPA), and the efficacy of this method was assessed based on encapsulation efficiency, active loading, and yield. Additionally, the physical and chemical properties of FCGA‐TOCO coacervates were assessed using thermogravimetric analysis, rheological measurements, and morphological examinations. These coacervates were then introduced into direct emulsions and compared with emulsions containing non‐encapsulated α‐tocopherol under storage to evaluate their impact on microstructure, texture, and stability of both emulsions and active.

## MATERIALS AND METHODS

### Materials

Fungal chitosan (FC) with a degree of deacetylation: 80.9 ± 0.1% (determined by conductimetric dosing) and an average molecular weight of 39 ± 1 kDa was acquired from Kraeber & Co GmbH (Ellerbek, Germany) and gum Arabic *Senegal* (GA) (average molecular weight: 393 ± 23 kDa was kindly provided by Alland & Robert [France]). Viscosity measurements were assessed to determine the mean molecular weights of both polymers, with Mark‐Houwink‐Sakurada parameters reported in the literature by Kasaai [28] for chitosan and by Gómez‐Díaz et al. [29] and Idris et al. [30] for gum Arabic. Glycerol and sweet almond oil were purchased from Aromazone (France), the diisopropyl adipate DUB DIPA (DIPA) was kindly provided by Stéarinerie Dubois (France), xanthan gum Rhodicare® T by Rhodia (France), glyceryl stearate Dracorin® CE by Symrise (Germany) and the preservative mixture Geogard 221, consisting of benzyl alcohol, dehydroacetic acid and aqua by Arxada AG (Switzerland). α‐tocopherol (TOCO) (≥ 95%) was purchased from Sigma Aldrich (Germany). Hydrochloric acid (HCl) and glacial acetic acid were purchased from Fisher Scientific (USA). Absolute ethanol (EtOH) was purchased from Brabant (France).

### Methods

#### Preparation of biopolymer stock solutions

Biopolymer solutions were prepared by dissolving FC in acetic acid (1% v/v) and GA in demineralised water at room temperature (RT) for 4 and 2 hours under magnetic stirring, respectively. The biopolymer solutions were kept overnight at 4°C to ensure complete polymer hydration. The concentration of biopolymer solutions was 5% and 10% w/w, respectively.

#### Stirring devices

In this study, two types of stirring devices were employed: magnetic stirring and mechanical stirring. The latter was conducted using a Rayneri Turbotest (VMI Mixing, France), equipped with a defloculator turbine of either 35 mm diameter for complex coacervation, or 55 mm diameter for emulsions. A digital rotor‐stator device Ultra‐Turrax T25 was utilised for high shear‐rates processes, involving a turbine S25N‐25F (IKA, Freiburg, Germany).

#### Preparation of α‐tocopherol complex coacervates (FCGA‐TOCO)

**FIGURE 1:**
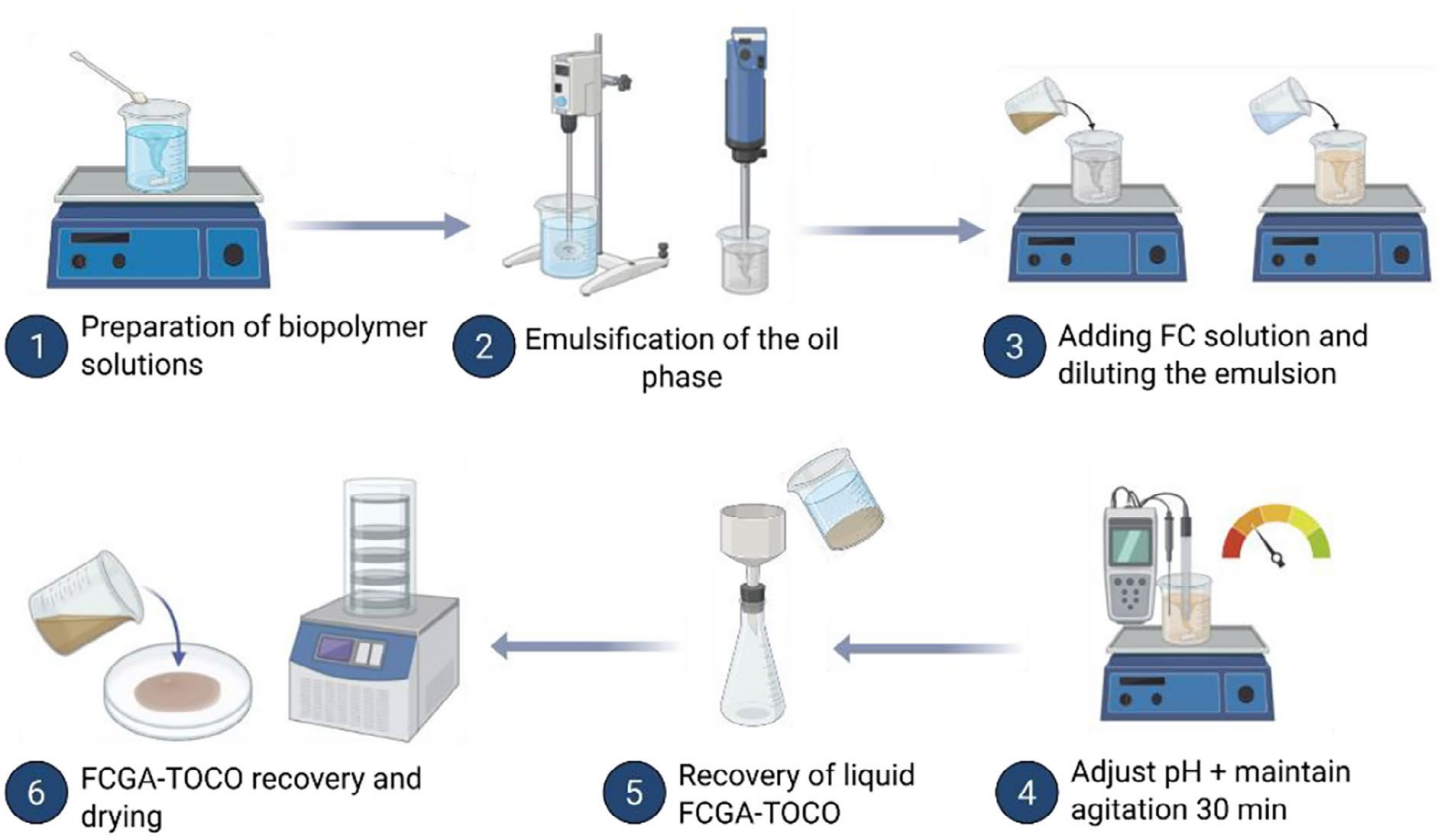
Protocol of encapsulation of α‐tocopherol by complex coacervation with fungal chitosan and gum Arabic (FCGA‐TOCO).

The FCGA‐TOCO coacervates were prepared by combining FC and GA in a polymer weight ratio of 1:4 and adjusting the pH to 4.8 (Figure 1). TOCO was dissolved in DIPA at a concentration of 50% w/w by magnetic stirring. The resulting solution was then emulsified in a solution of GA 5% w/w. This emulsification process was carried out using defloculator (35 mm) operating at 1000 rpm for 10 minutes. The resulting emulsion was then stirred using a rotor‐stator at a speed of 8000 rpm for an additional 5 minutes. The FC solution, with a concentration of 5% w/w, was gradually incorporated into the emulsion while stirring magnetically for 10 minutes. Subsequently, the entire FCGA‐TOCO solution was diluted to achieve a total solid content of 10% w/w and the magnetic stirring was continued for an additional 10 minutes. After adjusting the pH to the desired level of 4.8, the magnetic stirring of the solution was continued for another 30 minutes, and the resulting solution was left at rest at RT for 24 hours. Then, the polymer mixture was filtered by Büchner funnel using Whatman no. 42 filter paper. The liquid‐like coacervates were subsequently transferred to Petri dishes and subjected to freeze‐drying at a temperature of −96°C for 4 hours. The resulting powder was obtained by grinding the frozen coacervates.

The yield of the complex coacervates is defined as the ratio between the total mass of recovered particles (FCGA‐TOCO) and the theoretical mass of solid materials contained in the initial solution (i.e., FC, GA, and DIPA+TOCO) (Equation 1).

(1) $$\text{Yield}\;\left(\%\right)=\frac{m_{\text{FCGA}-\text{TOCO}}}{m_{\mathrm{FC}}+m_{\mathrm{GA}}+m_{\text{DIPA}+\text{TOCO}}}\times 100.$$

#### Formulation of α‐tocopherol emulsions

Emulsions containing α‐tocopherol were prepared using a previous direct emulsion (O/W) developed in the laboratory, selected for its long‐term storage stability, with an emphasis on natural and bio‐based ingredients. Two formulations were prepared: one with non‐encapsulated TOCO (OW‐TOCO) and another with FCGA‐TOCO coacervates (OW‐FCGA‐TOCO), to study the impact of the coacervates on the emulsion. OW‐TOCO and OW‐FCGA‐TOCO compositions are detailed in Table 1. A mixture of glyceryl stearate citrate and water was heated up to 78°C while mechanically stirred with a defloculator (55 mm diameter) until the surfactant completely dissolved. The oil phase, consisting of sweet almond oil (and non‐encapsulated TOCO for OW‐TOCO emulsions), was also heated to 78°C and then incorporated into the surfactant solution under continuous mechanical stirring (700 rpm) for 2 minutes. A rotor‐stator device was then used to form the emulsion at a speed of 9500 rpm for 2 minutes, and the emulsion was then cooled down to 35°C while being continuously stirred with a defloculator. FCGA‐TOCO coacervates (for the OW‐FCGA‐TOCO emulsion) were then introduced and mixed under continuous stirring at 700 rpm for an additional 5 minutes. Subsequently, xanthan gum was mixed with glycerin and incorporated into the emulsion for 10 minutes under continuous stirring at 700 rpm. Finally, the preservative was added to the emulsion and mixed for two additional minutes. The final emulsions were left at RT for 1 day and then transferred to appropriate containers for storage at 4°C prior to further testing and storage under accelerated aging conditions at 40°C. The stability of the emulsions was evaluated by monitoring changes in microstructure, particle size distribution, and rheological properties for 28 days at 40°C. Emulsions were prepared in duplicate to ensure repeatability.

**TABLE 1:** Detailed composition of the emulsions with non‐encapsulated (OW‐TOCO) and encapsulated TOCO (OW‐FCGA‐TOCO).

Ingredients (INCI)	Supplier	Function	%
Aqua	N.A.	Solvent	QSP 100
Glycerin	AromaZone	Humectant	3.0
Xanthan Gum	Rhodia	Texture agent	0.4
Glyceryl stearate citrate	Symrise	Surfactant	4.0
Prunus Amygdalus Dulcis Oil	AromaZone	Emollient	15.0
Benzyl alcohol, dehydroacetic acid, aqua	Arxada AG	Preservative	1.0
TOCO or FCGA‐TOCO	Sigma Aldrich	Antioxidant	0.28/1

Abbreviations: FCGA, fungal chitosan and gum Arabic; TOCO, α‐tocopherol.

#### Sample extraction

TOCO extraction from coacervates was carried out by weighing 100 mg of the sample in a plastic tube with 10 mL of EtOH. The mixture was placed in an ultrasonic bath (VWR Ultrasonicator, 120 W) at RT for 10 min to extract the encapsulated TOCO.

Extraction protocol of TOCO from emulsions was adapted from De Vaugelade et al. [31] as follows: 1 g of emulsion was weighed in a plastic tube with 10 mL of EtOH, then placed in an ultrasonic bath (VWR Ultrasonicator, 100 W) at 60°C for 45 min and finally centrifuged at RT at 936 g for 20 min; supernatant was collected, then filtered through a 0.45 μm cellulose acetate pore‐sized filter prior to LC‐UV analysis (in triplicate).

#### Determination of α‐tocopherol concentrations by liquid chromatography

The sample's TOCO concentration was determined using an Agilent 1200 system (Agilent Technologies, Waldbronn, Germany) equipped with a C18 XDB 5 μm 6.6 × 150 mm column and a UV–vis detector Agilent Infinity 1260 VL+. The compound was eluted at 40°C in isocratic mode using Milli‐Q water/MeOH at a ratio of 2/98 as a mobile phase for complex coacervates and 5/95 for emulsions at a flow rate of 1.5 mL/min with the detection wavelength set at 290 nm. The elution time of TOCO was constant at 4.7 min. A standard calibration curve (R^2^ = 0.999) was used to determine the TOCO concentration based on the chromatograph peak area.

The encapsulation efficiency (EE) was calculated as the relationship between the experimental concentration and the theoretical concentration of TOCO in the coacervates (Equation 2):

(2) $$\begin{array}{c} \mathrm{EE}\;\left(\%\right)=\frac{{\left[\text{TOCO}\right]}_{\exp}}{{\left[\text{TOCO}\right]}_{\mathrm{ini}}}\times 100. \end{array}$$

#### Optical microscopy observations

Emulsions and FCGA‐TOCO microstructures were examined using an optical microscope (ECLIPSE Ni‐U, Nikon) equipped with a camera under brightness. Images were obtained at a 200x magnification and analysed using Nikon software (NIS Element Viewer).

#### Particle size distribution

Particle size distribution of FCGA‐TOCO and emulsions was analysed using static light scattering with a SALD‐7500 nano laser diffraction particle size analyser equipped with a violet semiconductor laser (405 nm) and a reverse Fourier optical system (Shimadzu Co., Ltd., Japan). Samples were diluted in demineralised water to achieve an absorption parameter of 0.2 prior to any measurement. During measurement samples were continuously stirred in the batch cell to ensure homogeneity. For each product, three samples were collected and analysed, and measurements were performed in triplicate for each sample. Data analysis was done using Wind SALD II software and results were presented as average values of D10, D50 and D90 (μm). These values indicate the size below which 10%, 50% or 90% of all particles are present in the sample, respectively.

#### Rheological measurements

Rheological properties of the emulsions were evaluated by continuous and oscillatory measurements, using a hybrid DHR‐2 rheometer (TA Instruments, USA) equipped with a 40 mm diameter aluminium plate geometry at 20°C. Once loaded, the sample was left at rest for 60 s prior to any measurement. Flow properties were determined using continuous ramp tests, recording the viscosity value as the shear rate increased from 0.001 to 1000 s^−1^ (logarithmic mode) over 300 s. Oscillatory measurements were conducted at a constant frequency of 1 Hz with increasing strain from 0,01% up to 200% to determine the linear viscoelastic region. The frequency sweep ramp was conducted over a range from 0,01 to 100 Hz at a fixed strain within the linear viscoelastic region previously determined. Both storage (G') and loss (G") moduli were recorded to characterize samples' viscoelastic properties. Measurements were carried out in duplicate.

#### Thermal gravimetric analysis (TGA) and moisture content

The thermal characteristics of the FCGA‐TOCO coacervates and starting materials (5–10 mg) were assessed using a Setsys TGA 1200 (SETARAM, France), under an air atmosphere with a temperature ranging from 25°C to 600°C at 10°C/min. Moisture content was determined by measuring the total weight loss observed once a temperature of 125°C was attained.

#### Texture analysis of emulsions

Texture analyser TA.XT Plus (Stable Micro Systems, Cardiff, UK) was utilised to evaluate the consistency of OW‐TOCO and OW‐FCGA‐TOCO emulsions. Compression tests were performed at RT using the following method: 1 mL of sample was placed on the base of the device using a Microman® M250 GILSON and compressed using a cylindrical aluminium P/35 probe (35 mm). The compression was carried out up to a gap of 0.5 mm between the probe and the base at a constant speed of 1 mm/s before the probe returned to its initial position. The curve force = f(time) was recorded for each test and various parameters were collected, including the minimum and maximum forces (g) and the positive and negative areas (g/s).

#### Storage stability

The retention properties of FCGA‐TOCO coacervates were evaluated and compared to pure TOCO. One gram of coacervates and the corresponding amount of pure TOCO (0.3 g) were stored at 40°C for 56 days to assess their stability under accelerated conditions.

OW‐TOCO and OW‐FCGA‐TOCO emulsions were stored in closed containers at 40°C for 28 days. Stability over time was assessed by particle size analysis, optical microscopy observations, and frequency sweep tests. In addition, the remaining amount of TOCO was determined weekly according to the LC‐UV method previously described.

##### Statistical analysis

All analyses were carried out at least in duplicate, and results are displayed as mean ± standard deviation (SD). Collected data were analysed using one‐way ANOVA and compared with Tukey's HSD test, a post‐hoc method for identifying significant differences between group means, at a 95% confidence level, using XLSTAT software (Version 2012.1.01, Addinsoft, Paris, France).

## RESULTS AND DISCUSSION

### Production and characterisation of coacervates

FCGA‐TOCO coacervates were produced by complex coacervation using a mixture of FC and GA in a 1:4 (w/w) ratio at pH 4.8. The oil phase, which consists of a solution of 50%wt TOCO in DIPA, was introduced at a ratio of FCGA:core 1:1 (w/w) at RT. Microcapsules were then characterised before and after the coacervation process, with their corresponding properties detailed in Table 2.

**TABLE 2:** Characteristics of α‐tocopherol coacervates (fungal chitosan and gum Arabic‐α‐tocopherol) prepared by complex coacervation.

Yield (%)	Active loading (%)	Encapsulation efficiency (%)	Moisture content (%)
81.5 ± 5.0%	27.2 ± 1.9%	87.0 ± 2.5%	5.4 ± 0.1

Once complex coacervation process finished, 81.5% of the FCGA‐TOCO solid coacervates were recovered by freeze‐drying; resulting microcapsules contained 27.2% TOCO and had a moisture content of 5.4%. The stability of microcapsules is significantly affected by the moisture content, thus making it a crucial parameter. A reduced moisture content may prevent the active substance from being released, thus enhancing its stability [32]. The encapsulation efficiency, reflecting the proportion of effectively encapsulated TOCO compared to the initial amount introduced, was 87.0%. Although the combination of FC and GA has not been used elsewhere to encapsulate TOCO, some studies have shown high efficiencies when using chitosan from animal sources. Huang et al. [33] obtained an encapsulation efficiency of 56.3% by combining chitosan with soy protein isolate, while Budinčić et al. [34] achieved an encapsulation efficiency of 73.2% by combining it with sodium lauryl ether sulfate. Carpentier et al. [6] studied the TOCO encapsulation process using a combination of natural proteins and polysaccharides, including pea protein and different gums, namely tragacanth, Arabic and tara. Encapsulation efficiencies ranged between 42.7% and 77.4%, with the highest achieved when combining pea protein isolate with GA. However, it is challenging to compare with these studies as they used low TOCO concentrations (only few percent) and employed different recovery techniques such as spray‐drying. Through the integration of FC and GA via complex coacervation, we efficiently encapsulated TOCO with significantly greater yield and encapsulation efficiency compared to existing literature, producing surfactant‐free and highly concentrated microcapsules (27% TOCO).

### Morphology and particle size of coacervates

Figure 1 and Table 3 present the evolution of the morphology and particle size of particles before and after the coacervation process.

**TABLE 3:** Particle size of the gum arabic‐α‐tocopherol (GA‐TOCO) emulsion before coacervation and fungal chitosan‐gum arabic (FCGA) formed coacervates (in volume).

Name	Mean value (μm)	D10 (μm)	D50 (μm)	D90 (μm)
GA‐TOCO	3.4 ± 0.6	0.3 ± 0.1	5.6 ± 0.6	22.2 ± 10.8
FCGA‐TOCO	86.8 ± 3.5	42.3 ± 2.2	91.1 ± 3.5	168.8 ± 3.7

*Note*: D10, D50, and D90 represent the particle sizes beneath which 10%, 50%, and 90% of the particles are smaller, respectively.

**FIGURE 2:**
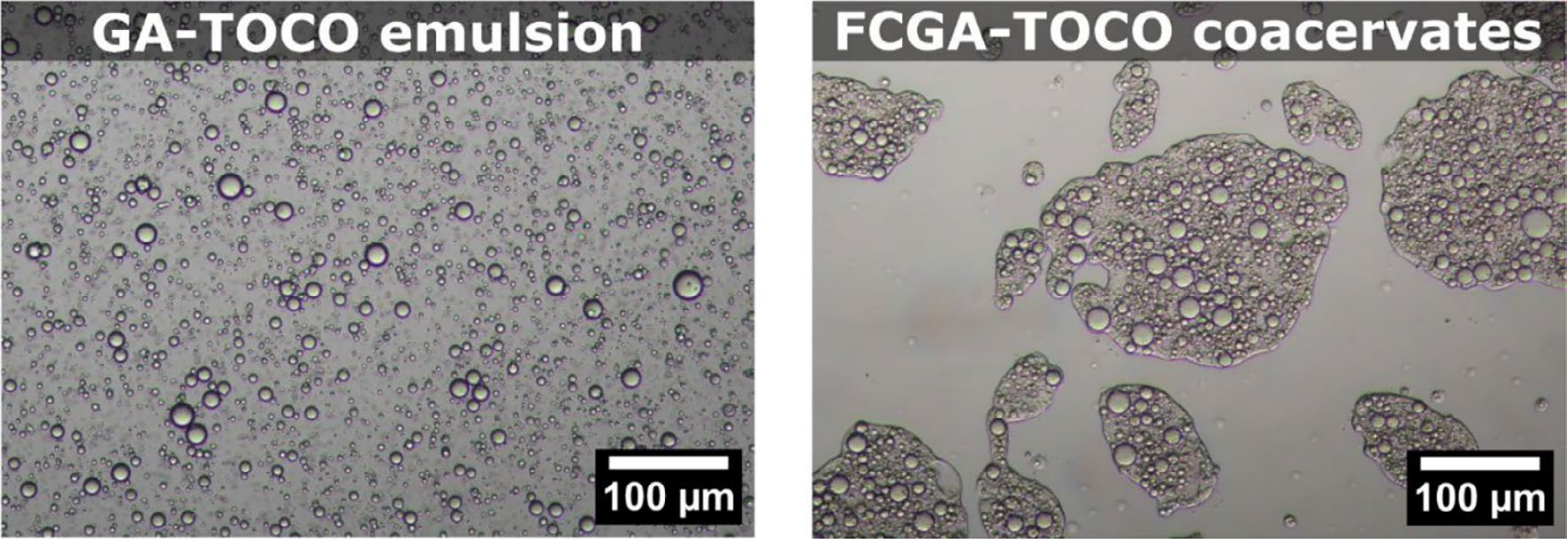
Observations of the gum arabic‐α‐tocopherol (GA‐TOCO) emulsion before coacervation and the formed α‐tocopherol coacervates (FCGA‐TOCO) by optical microscopy.

After emulsifying the oil phase (50%wt TOCO in DIPA) in the GA solution, droplets with an average diameter of 3.4 μm were observed. In contrast, FCGA‐TOCO coacervates showed a sharply different distribution with an average diameter of 86.8 μm. Optical microscopy showed that coacervates formed irregular shapes around the oil droplets in the complex coacervation process (Figure 2).

Dong and Cui [35] provided similar observations regarding the complex coacervation of proteins and GA and were attributed to the viscoelasticity of the coacervates. Liquid‐like complex coacervates quickly deposited on the oil droplets surface and sticked together, while solid‐like coacervates tended to form separate droplets. This is consistent with our previous studies, as FCGA‐TOCO coacervates exhibited liquid viscoelastic behaviours with high deformability properties (data not shown).

### Thermogravimetric analysis (TGA) and moisture content

**FIGURE 3:**
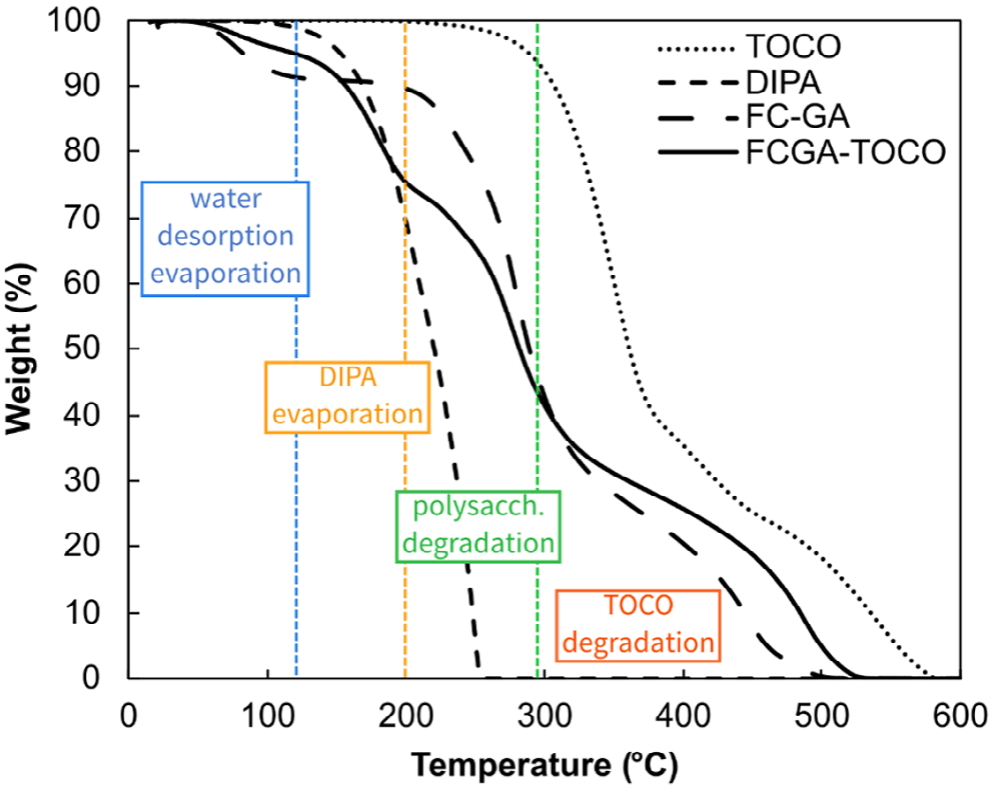
Thermal degradation profile of pure α‐tocopherol (TOCO), diisopropyl adipate (DIPA), empty coacervates (FC‐GA) and α‐tocopherol coacervates (FCGA‐TOCO).

Thermo‐oxidative stability of freeze‐dried FCGA‐TOCO coacervates, as well as pure TOCO and DIPA, was assessed using TGA analysis under air atmosphere, with a temperature ranging from 25 to 600°C. Corresponding thermograms are presented in Figure 3. The moisture content was determined by quantifying the total mass loss at 125°C.

Pure TOCO demonstrated high thermal stability, with the initial weight loss starting around 250°C. DIPA, selected as carrier oil, started to degrade around 130°C, which is related to its boiling point. FCGA‐TOCO coacervates exhibited four distinct stages of degradation. The first one, which takes place at temperatures below 125°C, is associated with the process of water desorption and evaporation, resulting in a weight loss of approximately 5.4%. For temperatures between 130 and 200°C, DIPA evaporation was observed; then, around 230°C, polysaccharides started to decompose [36, 37], followed by TOCO, with complete degradation observed at 530°C. TOCO microcapsules exhibited significant enhancement of thermal stability, characterised by a slower decomposition rate at temperatures exceeding 300°C, compared to FC‐GA coacervates. The mass loss of 28% occurring at temperatures below 220°C, was mainly attributed to the evaporation of DIPA while the other components seemed unaffected. Therefore, the designed complex coacervates can be considered thermally stable up to 220°C, the latter being the maximum temperature where TOCO and polysaccharides were not affected, and making them suitable for high‐temperature processes.

### Storage stability of complex coacervates

**FIGURE 4:**
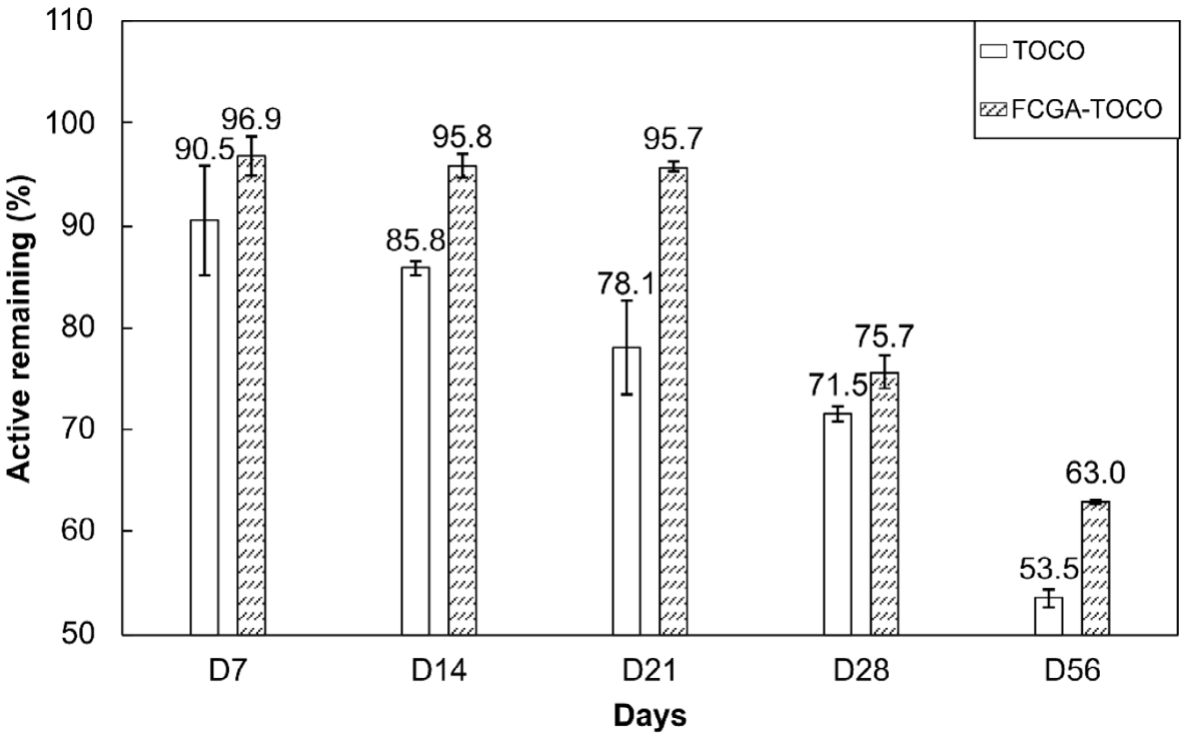
Stability of pure α‐tocopherol (TOCO) and complex coacervates (FCGA‐TOCO) over 56 days at 40°C.

To evaluate the stability of TOCO coacervates, microcapsules, and pure TOCO were stored at 40°C in closed containers. The remaining TOCO was quantified over 2 months by LC‐UV and corresponding results are presented in Figure 4.

Pure TOCO degradation at 40°C appeared faster than that of FCGA‐TOCO coacervates, particularly during the first 21 days of storage. During this period, a loss of 27.9% of pure TOCO was observed, while the FCGA‐TOCO coacervates remained constant, with 95.7% remaining after 3 weeks. Nevertheless, after 28 days, there was a significant reduction of 20% in TOCO for complex coacervates. This decrease continued over the next 28 days, resulting in a remaining TOCO level of 63%. On the contrary, only 53.5% of the initial TOCO concentration remained after 56 days in the same conditions. Despite the few studies conducted on the encapsulation of TOCO through complex coacervation, to our knowledge, the stability of encapsulated TOCO during storage has been poorly investigated [6, 33, 34]. In their study, Fang et al. [38] evaluated the stability of pure and encapsulated TOCO in whey protein isolate particles or emulsions. Despite the absence of pure TOCO after 1 month at 45°C, the produced particles were allowed to retain 38% of TOCO after 58 days. However, the fast degradation of the active ingredient could be attributed to the use of low active concentrations (0.06% and 0.12%, respectively). On the contrary, in the current work, complex coacervates were efficient in improving the stability of TOCO, even at high active concentrations. To summarize, these microcapsules enhance the retention and stability of the encapsulated compound over time, facilitate their incorporation in formulations, and improve the product's performance and longevity across various industries (food, cosmetics, pharmaceutical).

### Characterisation of emulsions containing α‐tocopherol

Two direct O/W emulsions were prepared, one with non‐encapsulated (OW‐TOCO) and the other with encapsulated α‐tocopherol (OW‐FCGA‐TOCO). After 24 hours of storage at RT, the particle size distributions, microstructure, and rheological properties were evaluated.

### Microstructural characterisation of emulsions

**FIGURE 5:**
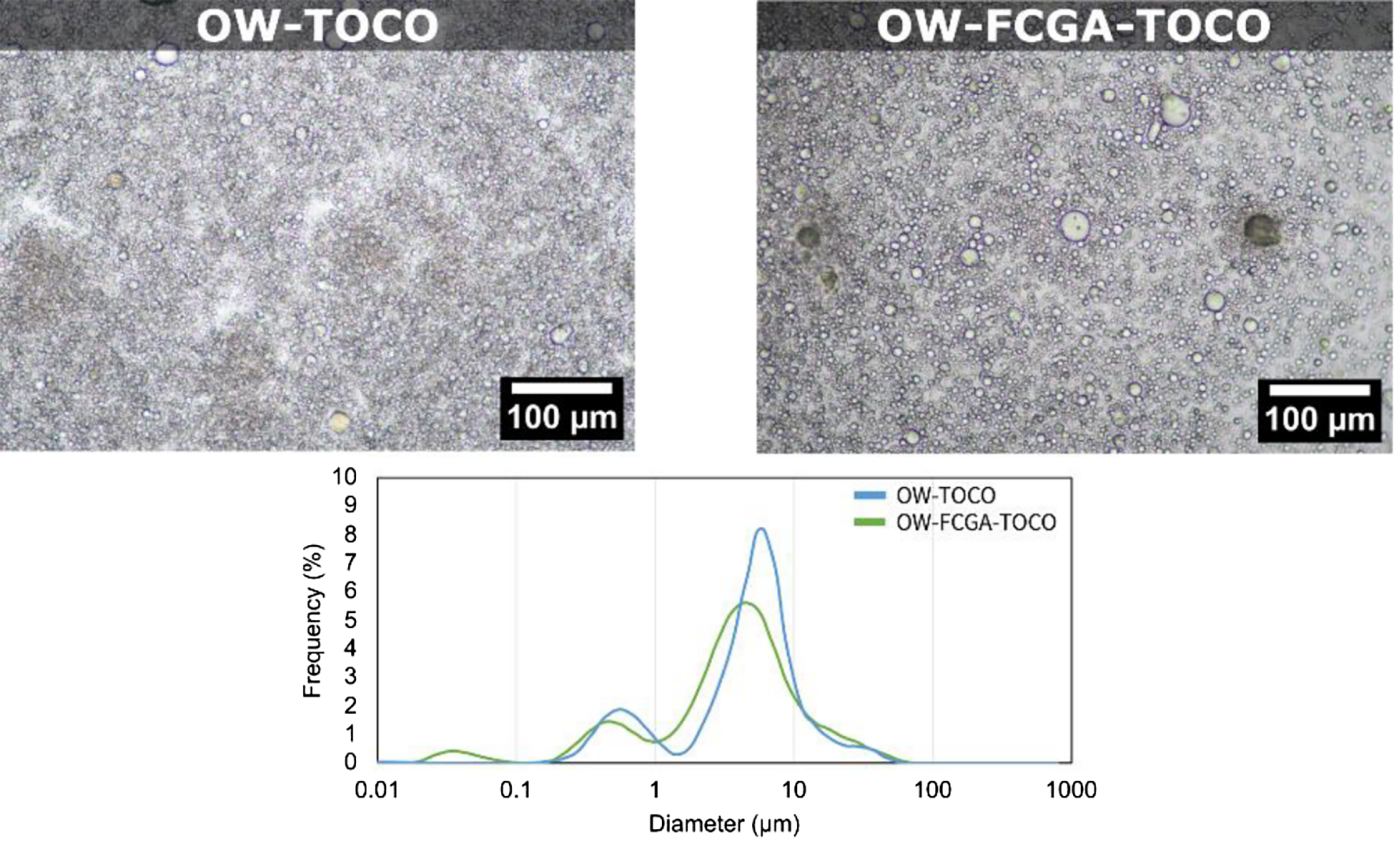
Optical microscopy observations and particle size distributions of non‐encapsulated (OW‐TOCO) and encapsulated (OW‐FCGA‐TOCO) α‐tocopherol emulsions.

Emulsions' granulometric characteristics were determined by SLS to assess the impact of the addition of coacervates on their size distribution and microstructure. Results presented in Figure 5 showed that both emulsions have a multimodal distribution with a mean diameter of 4.0 ± 0.5 μm for OW‐TOCO, and 6.8 ± 0.2 μm for OW‐FCGA‐TOCO the latter having a broader distribution around 1–100 μm. This may be due to both the TOCO/DIPA initial droplet incorporated in coacervates and the size of the coacervates themselves. Optical microscopy revealed oil droplets aggregating on the surface of complex coacervates. Coacervates being large particles, their compression between the microscope slides and coverslips may have generated the release of TOCO/DIPA droplets prior to their observation.

### Macrostructural characterisation of emulsions

**FIGURE 6:**
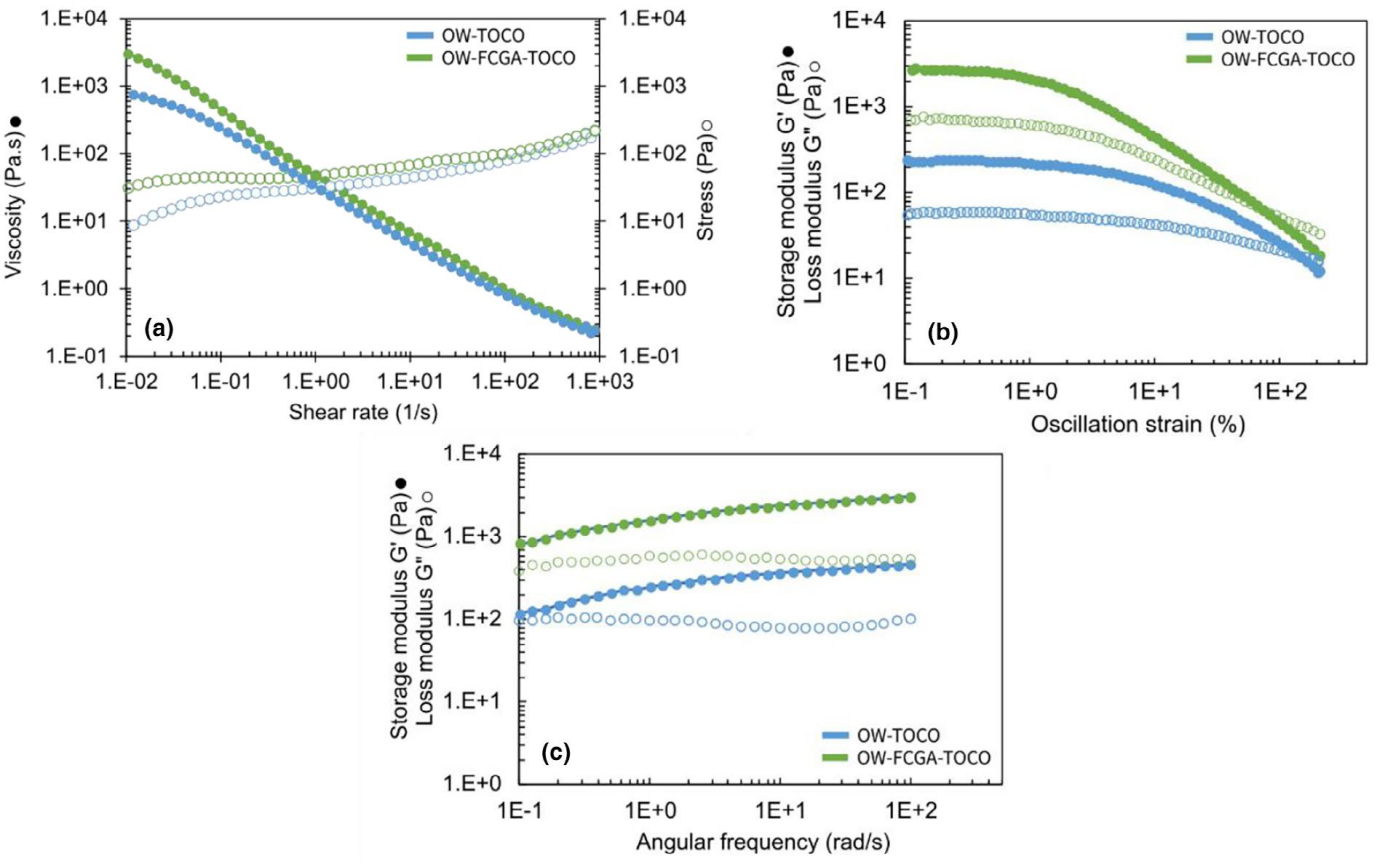
Rheological properties of non‐encapsulated (OW‐TOCO) and encapsulated (OW‐FCGA‐TOCO) α‐tocopherol in emulsions: Flow test (a), deformation test (b), and frequency sweep (c).

Rheological properties of emulsions are presented in Figure 6. Both emulsions displayed a shear‐thinning behaviour, with a significant viscosity increase at low shear rates in the presence of coacervates. Barnes [39] correlated the various shear gradients encountered by cosmetic cream emulsions with typical situations, including manufacturing, packing, storage, and skin application. Moreover, Gore et al. [40] also correlated rheological properties with in‐vivo spreading. Thus, the results obtained at low shear rates suggest that integrating complex coacervates results in a higher consistency without significantly impacting the spreading on the human skin (10^3^–10^4^ s^−1^). Furthermore, the presence of FCGA‐TOCO coacervates affected the linear viscoelastic region, leading to a decrease in critical strain from 1.3% to 0.6%, thus illustrating increasing to solid‐like behaviour. Figure 6c illustrates that the presence of complex coacervates did not affect the behaviour of the emulsion. Both emulsions showed little dependence on frequency, but a tenfold increase in the loss modulus was noted when complex coacervates were added. The OW‐FCGA‐TOCO emulsions exhibited higher overall moduli due to stronger interactions occurring between the coacervates and the formulation's compounds.

**FIGURE 7:**
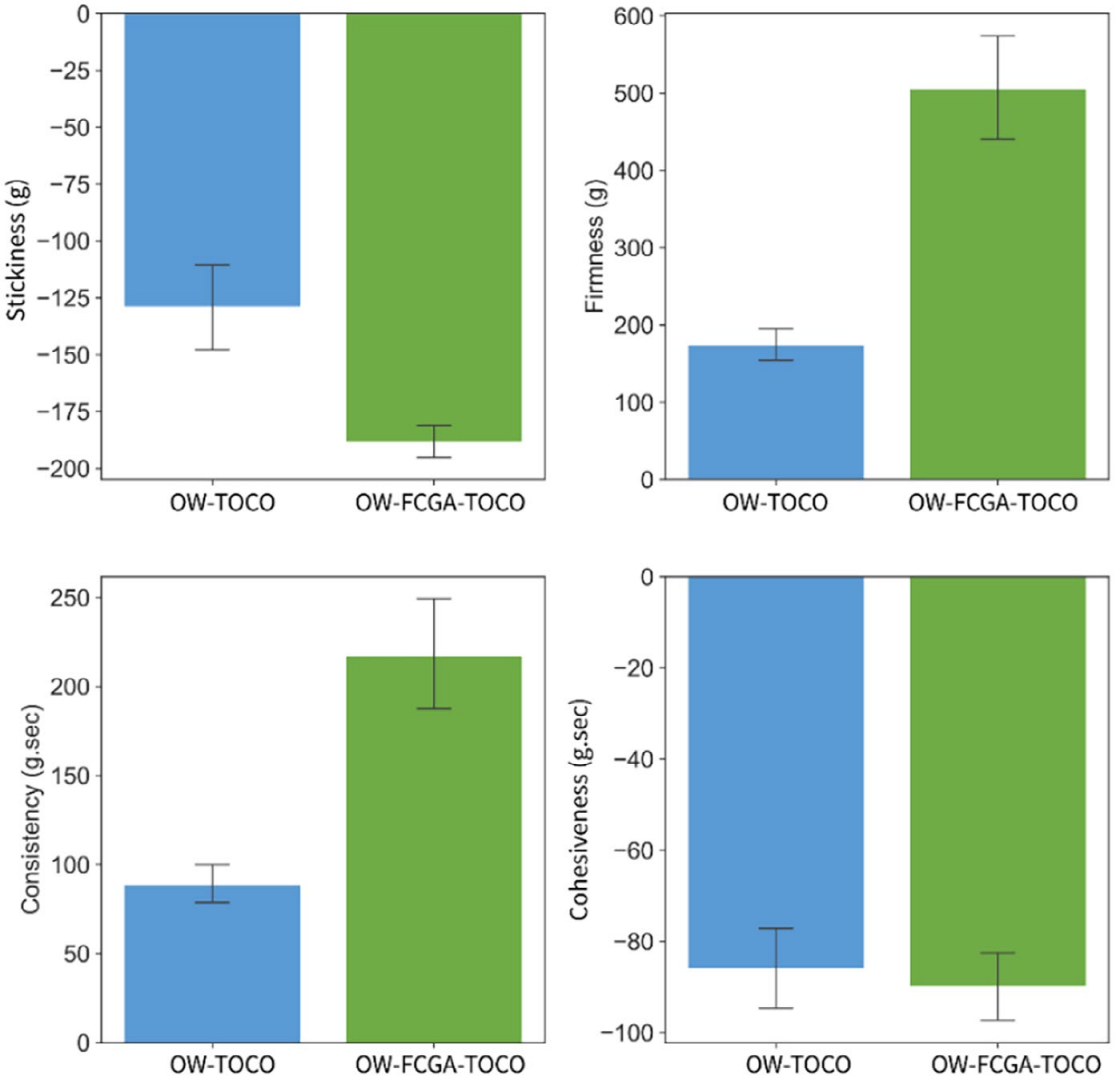
Textural properties of emulsions containing non‐encapsulated (OW‐TOCO) and encapsulated (OW‐FCGA‐TOCO) α‐tocopherol.

The textural properties play a crucial role in characterising emulsions applied topically and their sensory properties on human skin. Previous research highlights that instrumental assessment of textural properties is not only a valuable tool but also strongly correlated with a range of sensory attributes [41, 42]. Emulsions' texture analysis was performed through compression tests to evaluate their consistency, cohesiveness, stickiness, and firmness; as visible on Figure 7, incorporating FCGA‐TOCO coacervates into the formulation resulted in a significant increase in consistency and firmness, in accordance with their viscosity; in addition, a stickiness increase was observed for OW‐FCGA‐TOCO. Upon compression of the emulsion, coacervates flattened and stuck between the probe and the base, leading to an increase in the force required to detach the probe from the base. However, such a sticky effect does not reflect normal usage conditions, such as application on the skin. The cohesiveness, measured as the rate at which the probe returns to its initial position (g/sec), was not significantly impacted by the presence of complex coacervates. As a comparison, Böger et al. [27] observed a notable reduction in firmness and cohesiveness, while consistency and viscosity of O/W emulsions remained unaffected upon the incorporation of gelatin‐GA coacervates of coffee oil. In their study, microcapsules were previously dispersed in water at 50°C and incorporated in liquid form. Moreover, they attributed the enhanced spreadability to the roasted coffee oil, rather than to the existence of complex coacervates. Thus, depending on the textural properties desired, complex coacervates can be incorporated in both solid and liquid form.

### Stability of emulsions during storage

**FIGURE 8:**
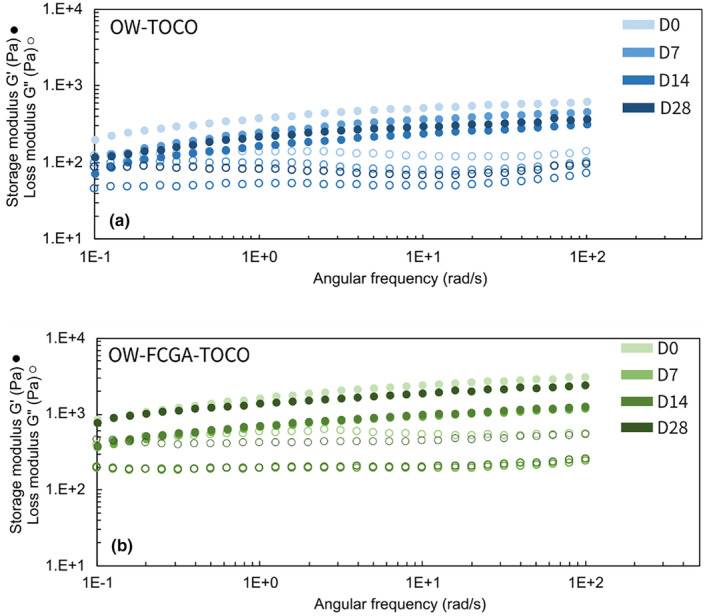
Frequency sweep of (a) non‐encapsulated (OW‐TOCO) and (b) encapsulated (OW‐FCGA‐TOCO) α‐tocopherol in emulsions over 28 days at 40°C.

**FIGURE 9:**
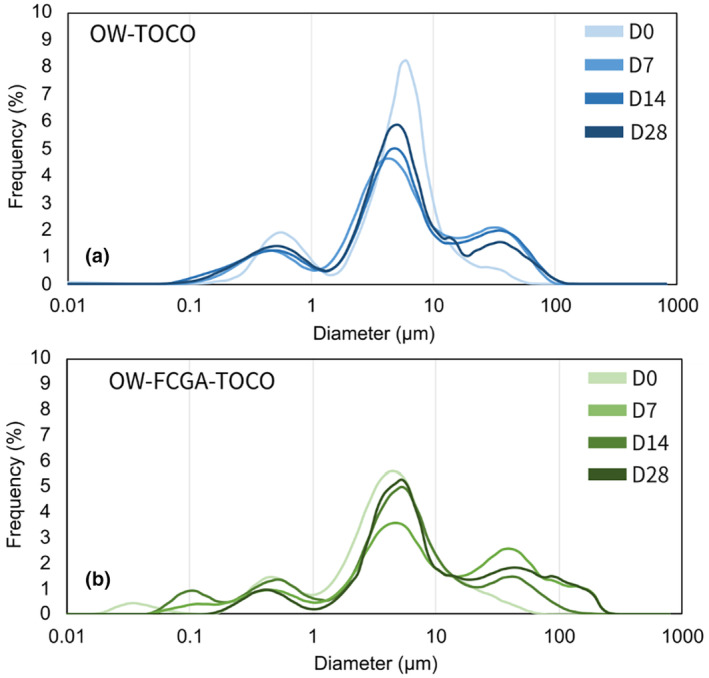
Particle size distribution of (a) non‐encapsulated (OW‐TOCO) and (b) encapsulated (OW‐FCGA‐TOCO) α‐tocopherol in emulsions over 28 days at 40°C. Results are displayed as volume diameters.

The OW‐TOCO and OW‐FCGA‐TOCO were put in sealed containers and stocked at 40°C for 28 days. Frequency sweep tests (Figure 8) and particle size analysis (Figure 9) were carried out to evaluate the stability of the emulsions. Although the organoleptic appearance of the emulsions remained unaffected, few microstructural alterations were observed. A slight decrease of both storage and loss modulus was observed for OW‐TOCO emulsions, correlated with the increase in the larger droplet population after 28 days observed in accelerated conditions (40°C).

However, microstructure variations of OW‐FCGA‐TOCO emulsions were observed during storage. Specifically, there was first a decrease in both the storage and the loss moduli over a period of 14 days at a temperature of 40°C, followed by an increase after 1 month. Over time, there was a significant increase in the larger particles fraction, as indicated by an increase in D90 (the particle size distribution where 90% of particles are smaller) from 13.2 to 72.3 μm. Different hypotheses can be proposed to try to explain this result: a phenomenon of coacervation, coalescence, or the increase in the state of aggregation. Therefore, xanthan gum being an anionic polysaccharide, it may interact with residual positive charges of FC to form larger aggregates [43]. Despite the challenging storage conditions at 40°C, both emulsions maintained a homogeneous appearance throughout the evaluation. Nevertheless, such minor changes in the microstructure are unlikely to significantly impact the sensory experience of consumers upon application.

**FIGURE 10:**
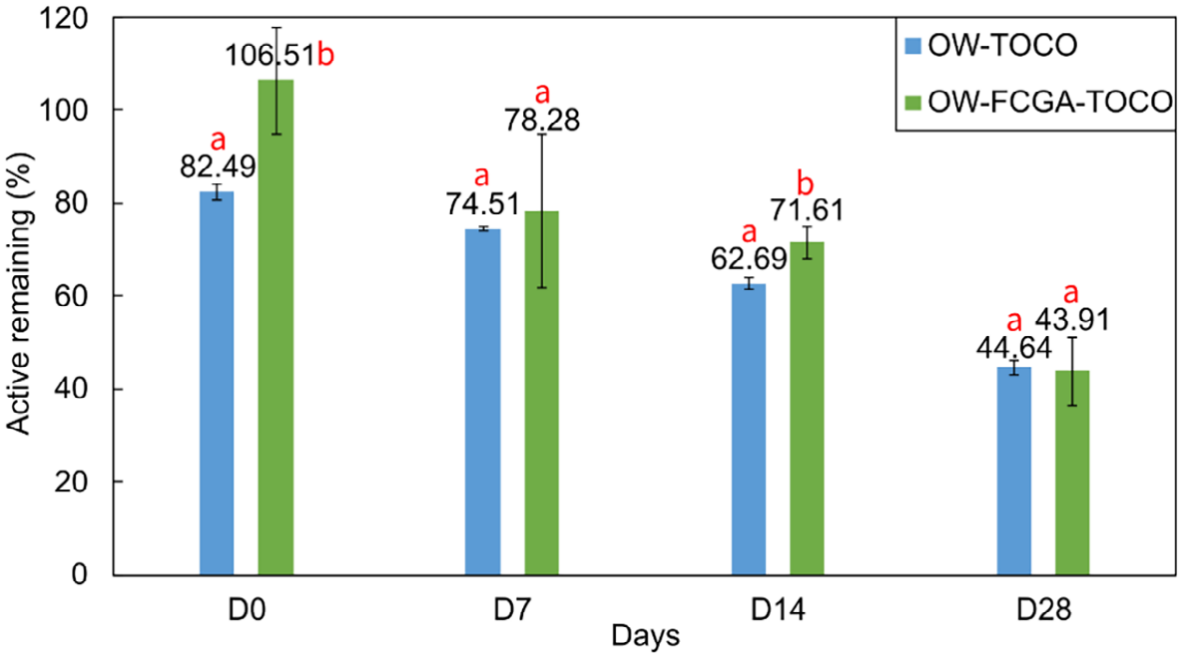
Stability of non‐encapsulated (OW‐TOCO) and encapsulated (OW‐FCGA‐TOCO) α‐tocopherol in emulsions over 28 days at 40°C. The values within the groups sharing different letters were significantly different (*p* < 0.05).

The remaining amount of TOCO in emulsions was evaluated during a 1‐month storage period at a temperature of 40°C (Figure 10). Following the formulation process, complete TOCO recovery was achieved from OW‐FCGA‐TOCO emulsions, while only 82.3% of the active ingredient was extracted from OW‐TOCO. This may be explained by the process utilised to prepare the emulsions; in fact, during the formulation of the OW‐TOCO emulsion, TOCO was first dissolved in the oil phase and then heated to a temperature of 78°C prior to its emulsification. A minimal amount of active substances may have undergone degradation during the heating process, leading to a decrease in recovery at D0. On the contrary, TOCO encapsulated in FCGA coacervates was protected against such a degradation when preparing emulsions. No significant variations were observed between the emulsions during the storage period, suggesting possible interactions between the complex coacervates and the matrix components. Prior observations indicated that complex coacervates improved the stability of TOCO; however, this stabilising effect was not visible in the emulsions. The complex coacervates probably interacted with the matrix components, possibly triggering the premature release of TOCO and reducing its effectiveness in protecting against external factors. In their study, Vuillemin et al. [44] established that temperature (ranged from 5 to 45°C) influenced the complex coacervation of chitosan and GA by potentially altering the conformation of chitosan, leading to reduced electrostatic interactions. Due to the complexity of emulsions and complex coacervates, additional research is needed to fully understand the phenomena taking place and develop stable cosmetic formulations.

## CONCLUSION

This study highlights the potential of fungal chitosan and gum Arabic as key ingredients in encapsulating sensitive lipophilic actives such as α‐tocopherol by complex coacervation. First, encapsulation of 50% α‐tocopherol in diisopropyl adipate produced coacervates approximately 90 μm in size with a very good encapsulation efficiency of 87%. These coacervates were demonstrated as thermally stable up to 220°C. In addition, encapsulated α‐tocopherol demonstrated greater stability during storage, showing a slower degradation rate compared to pure α‐tocopherol, with 63% of the active remaining after 2 months at 40°C. Corresponding innovative microcapsules, once incorporated into oil‐in‐water emulsions, allowed significantly enhancing the textural properties, which are crucial for topical applications where texture and sensory properties are essential. Furthermore, complex coacervates also enhanced the rheological and microstructural properties of the emulsions, leading to increased viscosity and viscoelasticity. Although emulsions remained stable after 1 month at 40°C, further research is essential to fully understand the destabilisation mechanisms and to evaluate the release kinetics of the active ingredient through the skin. Beyond preservation, these complex coacervates improve the formulations processability by smoothly incorporation into oil‐in‐water emulsions, thereby greatly enhancing their textural, rheological and microstructural characteristics. Moreover, chitosan being recognised for its antioxidant and antimicrobial activities, these multifunctional microcapsules may be suitable across a wide range of products. To summarize, these findings clearly highlight the potential of complex coacervation as a versatile and sustainable method for developing high‐performance formulations, contributing to the creation of innovative and eco‐friendly cosmetic delivery systems with enhanced functional properties.

## CONFLICT OF INTEREST STATEMENT

The authors have no conflict of interest to declare.
